# Monomorphic post-transplant lymphoproliferative disorder of the tongue: case report and review of literature

**DOI:** 10.1186/1746-1596-2-49

**Published:** 2007-12-19

**Authors:** Luis F Gonzalez-Cuyar, Fabio Tavora, Allen P Burke, Christopher D Gocke, Ann Zimrin, John J Sauk, Xiafeng F Zhao

**Affiliations:** 1 Department of Pathology, University of Maryland School of Medicine, 22 South Greene Street, NBW64, Baltimore, Maryland, USA; 2 Department of Medicine, University of Maryland School of Medicine, 22 South Greene Street, NBW64, Baltimore, Maryland, USA; 3 Department of Oral Pathology, University of Maryland School of Medicine, 22 South Greene Street, NBW64, Baltimore, Maryland, USA; 4 Departments of Pathology and Oncology, Johns Hopkins School of Medicine, Baltimore MD, USA

## Abstract

**Background:**

Post-transplant lymphoproliferative disorder (PTLD) is a spectrum of hematological diseases arising in context of immunosuppression after organ transplantation. PTLD can involve any organ; however, it is extremely rare in oral cavity.

**Methods:**

Using morphologic and immunophenotypic approaches we have studied a case of monomorphic PTLD of the tongue that developed in a patient following unilateral kidney and pancreas transplantation on immunosuppressive therapy. Additionally, cases of PTLD in the oral cavity were reviewed in the English literature.

**Results:**

The neoplasm showed large cell morphology and B-cell phenotype. In situ hybridization for Epstein-Barr virus was positive. Complete remission was obtained after decreasing immunosuppressive therapy. The patient remained in remission at 790 days' follow up.

**Conclusion:**

This rare case increased our awareness of PTLD in the oral cavity of patients following solid organ transplantation and immunosuppressive therapy.

## Introduction

Post-transplant lymphoproliferative disorder (PTLD) is a well-recognized complication after solid organ or bone marrow transplantation. It comprises a spectrum of pathologic patterns ranging from reactive Epstein-Barr virus (EBV)-driven lymphocytic/plasmacytic hyperplasia to high-grade malignant lymphomas [1]. PTLD may involve the lymph nodes or extranodal tissue at any site, including the organ allograft [2]. A series of 90 PTLD cases occurring in 4283 solid organ transplantations over a nine-year period revealed that two thirds of the patients presented with disease in a single site [3], and none of the cases presented in the tongue at diagnosis. Here we report a rare case of monomorphic PTLD of the tongue in a patient after kidney and pancreas transplantation, which was effectively treated solely by reduction of immunosuppression. Review of the English literature reveals only a few such cases that involve oral cavity (Table 1).

**Table 1:** Literature review of Posttransplant lymphoproliferative disorders (PTLD) arsing in the oral cavity

**Author**	**Age & sex, site**	**Onset**	**Diagnosis, site**	**EBV status**	**Treatment**	**Follow up**
**Doak et al 1968**	34 ♂, kidney	180 days	Reticular Cell Sarcoma, tongue & oral cavity	Viral inclusions	Antimycotic Medication NOS	Died 30 days after
**Staddlemann et al 1996**	65 ♀, kidney	600 days	NK/T-cell lymphoma, pharynx	Positive	Cyclosporine d/c, Four cycles CHOP	Remission for 1080 days
**Reams et al 2003**	16 ♀, R + L lung	330 days	Polymorphic B-cell lymphoma, tongue	Negative	Four cycles of Rituximab	Patient died of Sepsis
**Rolland et al 2004**	60 ♂, heart	3600 days	Peripheral T-cell lymphoma NOS, gingiva	Positive	Azathioprine d/c, decrease Cyclosporin	Remission for 360 days
**Rolland et al 2004**	61 ♂, heart	1410 days	DLBCL, maxilla	Posotive	Stepwise decrease in Ciclosporin	Recurrences at 180 and 510 days
**Bruce et al 2006**	45 ♀, pancreas	660 days	DLBCL, tongue	Positive	Sirolimus d/c Tacrolimus adjusted	Remission 660 days, AR
**Gonzalez-Cuyar et al 2007**	47, ♂, kidney pancreas	720 days	DLBCL, tongue	Positive	Decreased immunosupression	Remission for 790 days

## Case presentation

**Figure 1:**
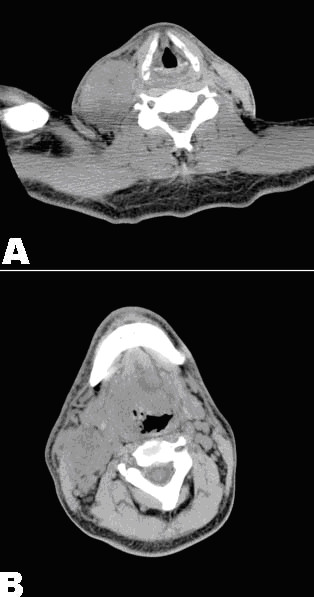
Images of computed tomography (CT) without contrast. **A**. Lesion at the base of tongue (indicated by an arrow); **B**. Conglomerate mass invading the right sternocleidomastoid muscle (indicated by an arrow).

**Figure 2:**
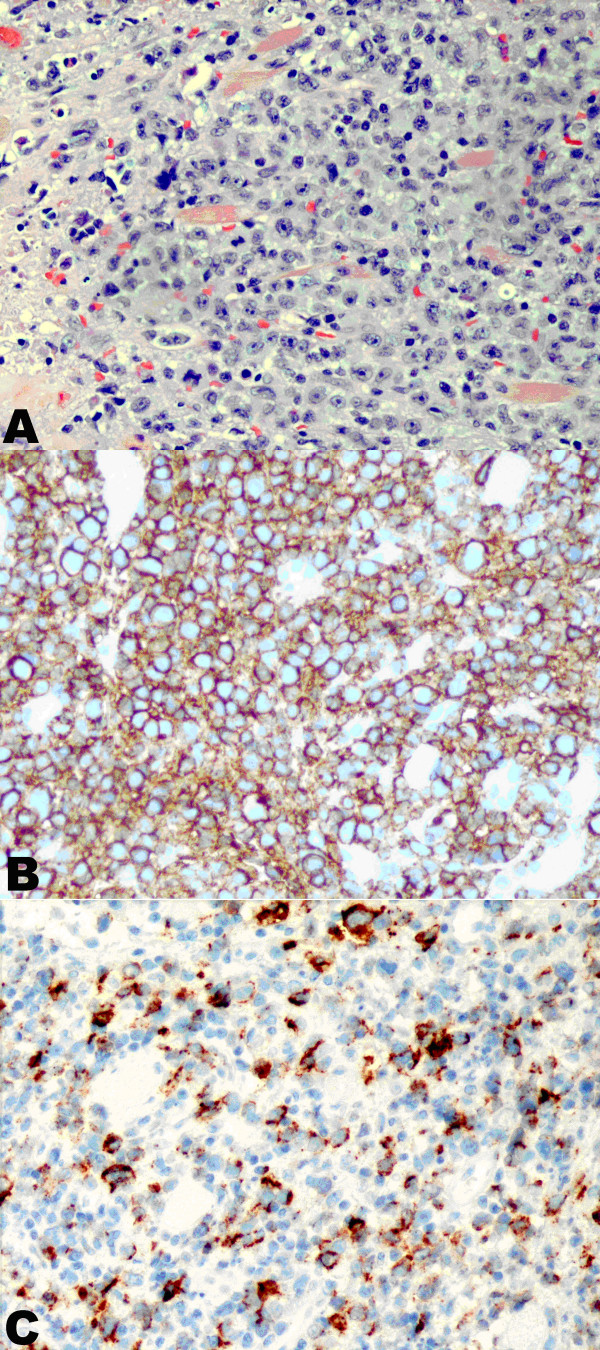
Microphotographs of the monomorphic PTLD. **A**. Large lymphoid cells with abundant cytoplasm, vesicular nuclei and prominent nucleoli (H&E, 400× magnification); **B**. Neoplastic cells positive for CD20 (immunoperoxidase stain, 400× magnification); **C**. Neoplastic cells positive for EBV LMP (immunoperoxidase stain, 400× magnification).

A 47-year-old Caucasian male with history of Diabetes Mellitus type I underwent simultaneous pancreas-kidney transplantation two years prior to the current admission. Past medical history also included depression and 20 pack-year smoking with cessation 10 years ago. At admission he presented with sore throat, progressive dysphagia, odynophagia, and weight loss. His immunosuppression regimen included tacrolimus (8 mg/day) and prednisone (5 mg/day). The physical exam was remarkable for a 4 cm exophytic lesion at the right base of tongue that appeared to extend to the inferior pole of the tonsillar fossa, as well as marked right cervical lymphadenopathy. Computed tomographic (CT) scan of the neck revealed an ill-defined mass in the right peripharyngeal region at the base of the tongue with deviation of the airway to the left (Figure 1A). In addition, a conglomerate mass representing a group of involved lymph nodes invading the right sternocleidomastoid muscle was also identified (Figure 1B). CT scan of the abdomen showed enlarged right mesenteric lymph nodes and an area of thickening of the small bowel wall. A panendoscopy with biopsy of the right base of the tongue lesion was performed. The hematoxylin-eosin (H&E) stained sections showed sheets of monotonous large atypical lymphoid cells with abundant cytoplasm, large vesicular nuclei and occasional prominent nucleoli (Figure 2A). These neoplastic cells infiltrated the adjacent skeletal muscles of the pharynx. Mitotic figures were rare. Large foci of necrosis were noted. Immunohistochemical stains demonstrated that the large cells were positive for CD20 (Figure 2B), CD30, BCL2, CD38 and EBV latent membrane protein (LMP) (Figure 2C). CD5 was aberrantly expressed in these cells. These cells were negative for CD3 and CD79a. Staining for immunoglobulin light chains kappa and lambda revealed only a few scattered polyclonal plasma cells. Molecular studies revealed *IGH *gene rearrangement in the neoplastic cells. Given the patient's history of solid organ transplantation and immunosuppression, it was diagnosed as monomorphic PTLD, consistent with diffuse large B-cell lymphoma. A CT scan of the pancreas and kidneys was negative for tumor. Repeated bone marrow biopsies were also negative for lymphoma involvement.

The immunosuppressive agents were withdrawn and the patient was closely followed. Ten weeks after the cessation of immunosuppressive therapy, the oral lesion had completely regressed and repeated CT scans of the head, neck and abdomen were constantly negative. Subsequent biopsies of the oral cavity were also negative. The patient has been followed for over 790 days and he currently remains in remission on lower doses of immunosuppressants, and with functioning allografts.

## Discussion

We have presented a rare case of monomorphic PTLD of the tongue with a complete remission after withdrawal of immunosupression. Through this case, we have also illustrated another successful example of managing the immunodeficiency-associated malignant lymphomas.

Recognition of PTLD as a clinical entity can be traced back to the mid 20^th ^century when the association between host immune response and immunosuppressive therapy with neoplasia was suggested, specially after solid organ transplantation [4-6] However, the development of hematological malignancies following transplantation of solid organs attributed to immunosuppressive therapy was first described by Doak et al [7] in 1968. In this report Doak and colleagues presented a 34 year-old man with history of renal transplantation who was treated with azathioprine and prednisone. Six months after the transplant, the patient presented with oral ulcers, which grew Candida species, and were initially treated with antimycotic medication. He died a month thereafter. Autopsy revealed multiple oral cavity, tongue, esophageal, and hepatic lesions that were composed of polygonal lymphoid cells with scant pale cytoplasm and large hyperchromatic nuclei. These were associated with occasional foci of necrosis, multinucleation, viral inclusions, macronucleoli and mitoses. At the time, it was referred to as reticular cell sarcoma.

In 1969 Penn et al [5] reported a case of a renal transplant patient on azathioprine and prednisone who developed a rapidly progressive left hemiparesis. Brain biopsy revealed a tumor of lymphoid origin. The patient was given radiotherapy with synchronous dosage reduction of the immunosuppressive therapy. He demonstrated marked neurologic improvement with shrinkage of the mass. Two decades later Starlz et al [8] first used the term post-transplant lymphoproliferative disorder (PTLD) and suggested that reduction and/or discontinuation of immunosupressive medications could lead to regression of the post-transplant malignancies.

Stadlmann et al [9] presented a 65-year-old male patient who had undergone kidney transplant in March of 1996. Twenty months later, he developed a small ulcer on the posterior aspect of the pharynx. The patient was serologically positive for EBV, and was on cyclosporine, prednisolone and azathriopine. Biopsy of the ulcer was diagnosed as a posttransplant associated NK/T cell lymphoma. Cyclosporine was subsequently discontinued and 4 cycles of chemotherapy with CHOP (cyclophosphamide, doxorubicin, vincristine and prednisone) were given. A three years' follow up revealed that the patient was still in remission.

In 2003, Reams et al published a series of 400 recipients of lung and/or heart transplant; among them 10 patients developed PTLD. Of particular interest was a 16-year-old female patient who had a history of cystic fibrosis status post bilateral lung transplantation and who was negative for EBV. Her immunosuppressive regimen consisted of cyclosporine A, azathioprine and metylprednisolone. Three-hundred and thirty days after the transplant the patient developed a lesion in the left base of tongue. She was diagnosed to have a polymorphic B-cell PTLD. After 4 cycles of rituximab, the patient died of sepsis.

In 2004 Rolland et al [10] reported two cases of oral PTLD in two patients with heart transplantation. The first patient was a 60-year-old male who was immunosupressed with azathriopine, cyclosporin, and prednisolone. During the first ten years following transplantation he had multiple episodes of gingival swelling that were attributed to cyclosporin. Biopsy of the gingiva demonstrated EBV-driven peripheral T-cell lymphoma, NOS. Azathrioprine was discontinued and cyclosporin was decreased. The patient was in remission for one year after the immunosupression adjustment. The second patient was a 61-year-old man who was on azathriopine, cyclosporin and prednisolone, and developed swelling of the maxilla and gingiva. Biopsy showed an EBV-driven diffuse large B-cell lymphoma with focal plasmablastic differentiation. The immunosuppressive therapy was adjusted and subsequently had two recurrences at 6 and 17 months post transplantation, which were treated effectively by stepwise decrease of cyclosporin.

Bruce et al [11] in 2006 presented a case of a 45-year-old female patient who was status post pancreatic transplantation and on an immunosuppressive regimen consisting of sirolimus, tacrolimus, and prednisone for 22 months. She presented with an ulcer on the ventral aspect of the tongue. Differential diagnosis of the lesion included granuloma of the tongue, infection and drug induced ulceration. Sirolimus was discontinued, tacrolimus was adjusted and a superficial biopsy was obtained. The biopsy revealed a traumatic granuloma with rare EBV+ lymphocytes by in-situ hybridization (EBER). Subsequently the entire lesion was resected and diagnosed as monomorphic EBV-associated PTLD, diffuse large B-cell lymphoma. The patient was followed for two years and was still in remission, but developed chronic rejection of the allograft.

Transplant recipients have a five-fold increased risk of developing *de novo *head and neck malignancies when compared to general population [12,13], of which the most common neoplasms are skin cancers [13,14] In the transplant population the incidence of PTLD is approximately 2%[11,15]. PTLD is a spectrum of lymphoproliferative disorders in patients who have undergone solid organ or bone marrow transplantation in the setting of immunosuppression [16], with EBV implicated in 2–5 % of adult patients [3] and of up to 20% of pediatric patients [16]. In the latter population it has been suggested that EBV-associated adenotonsilar enlargement could be a precursor to PTLD [17]. On average, patients have been diagnosed 2 years after transplantation [13]. In these patients, immunosuppression decreases the immunosurveillance of EBV-specific T-cells; and thus allows subsequent proliferation of EBV-infected B-cells [18-20]. Early development of PTLD is often associated with EBV infection and thus responds to reduction in immunosupression. However, later onset PTLD does not respond to reduced immunosuppression [20]. Later onset EBV-negative PTLD has also been reported and generally has a more aggressive clinical course [18-20].

The organs involved by PTLD vary depending on the difference in immunosuppressive regimens. The allograft itself is involved in only 25% of the cases [19]. Patients treated with tacrolimus often develop nodal and gastrointestinal PTLD [2,18]. In our patient, PTLD developed in the tongue as well as the possible lymph node (cervical and mesenteric) and gastrointestinal tract (as demonstrated by CT scan). Although the oral cavity may be regarded as the uppermost part of the gastrointestinal tract, reports of PTLD involvement of the tongue are extremely rare.

Initial treatment for PTLD is to reduce immunosupression [1]. A response is usually seen within 2–4 weeks of withdrawal of immunosupression [21], and reduction in immunosupression alone leads to long-term disease-free remission in 25–73% of adults [15,22]. The chances of complete remission seem to be directly related to the degree of differentiation of the neoplasm. Early and infectious mononucleosis-like lesions tend to regress more often with reduction in immunosupression alone, compared to monomorphic PTLD. A proportion of cases of both types, however, requires chemotherapy [23]. The benefit of withdrawing immunosupression and the risk of transplant rejection need to be carefully reconciled. The institution of chemotherapy also brings inherent risks of infections and *de novo *malignancies. Antiviral agents seem to have some effects on the early hyperplasic lesions [24,25]. However, these agents do not have significant effects on the lesion once monoclonality has emerged [26].

Although lymphoid malignancies of the tongue and oral cavity have previously been reported [27-30], complete remission of diffuse large B-cell lymphoma after withdrawal of immunosupression has rarely been documented, particularly in cases of PTLD. A consensus in the treatment of PTLD with maximum safety to the organ allograft is yet to be attained. However, the current report demonstrates the efficacy of reduction of immunosupression in the management of some cases of EBV-driven PTLD, even in the form of a high-grade diffuse large B-cell lymphoma.

Another common dilemma is differentiation opportunistic infections and PTLD. Patients with immunosupression after organ transplantation are at increased risk of infections and sepsis [31]. However, it should be noted that opportunistic infections with positive tissue and/or blood cultures might mask an underlying hematological malignancy [4,6,7,23]. Since immunosupressed patient are at increased risk of developing opportunistic infections and hematological malignancies, both possibilities need to be considered on the differential diagnosis. This case report stresses the importance of obtaining biopsies of oral cavity lesions in immunosuppressed patients following solid organ transplantation to direct appropriate treatment in a expeditious fashion.
